# Poly-ε-Caprolactone/Gelatin Hybrid Electrospun Composite Nanofibrous Mats Containing Ultrasound Assisted Herbal Extract: Antimicrobial and Cell Proliferation Study

**DOI:** 10.3390/nano9030462

**Published:** 2019-03-20

**Authors:** Raghavendra Ramalingam, Chetna Dhand, Chak Ming Leung, Hariharan Ezhilarasu, Praseetha Prasannan, Seow Theng Ong, Sundarapandian Subramanian, Mohammed Kamruddin, Rajamani Lakshminarayanan, Seeram Ramakrishna, Navin Kumar Verma, Kantha Deivi Arunachalam

**Affiliations:** 1 Center for Environmental Nuclear Research, SRM Institute of Science and Technology, Kattankulathur Campus, Kancheepuram, Tamilnadu 603203, India; raghavendra8025@gmail.com; 2 Department of Physics and Nanotechnology, SRM Institute of Science and Technology, Kattankulathur Campus, Kancheepuram, Tamilnadu 603203, India; 3 Center for Nanofibers and Nanotechnology, Department of Mechanical Engineering, Faculty of Engineering, 2 Engineering Drive 3, National University of Singapore, Singapore 117576, Singapore; hharan.arasu95@gmail.com; 4 Anti-Infectives Research Group, Singapore Eye Research Institute, The Academia, 20 College Road, Discovery Tower, Singapore 169856, Singapore; chetna.dhand@seri.com.sg; 5 Ophthalmology and Visual Sciences Academic Clinical Program, Duke-NUS Graduate Medical School, Singapore 169857, Singapore; 6 Department of Biomedical Engineering, National University of Singapore, Singapore 117581, Singapore; cm.leung93s@gmail.com; 7 Lee Kong Chian School of Medicine, Nanyang Technological University Singapore, Experimental Medicine Building, 59 Nanyang Drive, Singapore 636921, Singapore; praseethap@ntu.edu.sg (P.P.); st.ong@ntu.edu.sg (S.T.O.); 8 Department of Anatomy, SRM Medical College Hospital and Research Centre, Kattankulathur Campus, Kancheepuram, Tamilnadu 603203, India; sssspandian@gmail.com; 9 Materials Physics Division, Material Science Group, Indira Gandhi Centre for Atomic Research, Kalpakkam, Tamilnadu 603102, India; m.kamruddin@gmail.com; 10 Skin Research Institute of Singapore, 8A Biomedical Grove, #06-06 Immunos, Singapore 138648, Singapore

**Keywords:** electrospun hybrid mats, ultrasound assisted extraction, anti-infective wound dressing, poly-ε-caprolactone, gelatin

## Abstract

Electrospun fibers have emerged as promising materials in the field of biomedicine, due to their superior physical and cell supportive properties. In particular, electrospun mats are being developed for advanced wound dressing applications. Such applications require the firers to possess excellent antimicrobial properties in order to inhibit potential microbial colonization from resident and non-resident bacteria. In this study, we have developed Poly-ε-Caprolactone /gelatin hybrid composite mats loaded with natural herbal extract (*Gymnema sylvestre*) to prevent bacterial colonization. As-spun scaffolds exhibited good wettability and desirable mechanical properties retaining their fibrous structure after immersing them in phosphate buffered saline (pH 7.2) for up to 30 days. The initial burst release of *Gymnema sylvestre* prevented the colonization of bacteria as confirmed by the radial disc diffusion assay. Furthermore, the electrospun mats promoted cellular attachment, spreading and proliferation of human primary dermal fibroblasts and cultured keratinocytes, which are crucial parenchymal cell-types involved in the skin recovery process. Overall these results demonstrated the utility of *Gymnema sylvestre* impregnated electrospun PCL/Gelatin nanofibrous mats as an effective antimicrobial wound dressing.

## 1. Introduction

The annual incidence of skin injuries in developed countries was estimated to be around 4 million, which could be even higher in developing countries [1]. Damage to the skin can be caused by fire, accident, surgeries, acute trauma, genetic disorders, and chronic wounds [2]. Post-injury, the skin has the ability to repair itself and restore original function through a complex cascade of biochemical and cellular events. However, in case of critical wounds, it requires external support in the form of wound dressings to accelerate the healing process and restore normal integrity [3].

One of the major reasons for the delayed wound healing is an infection caused by bacterial colonization on the wound bed [4,5]. Bacteria have the ability to attach to the wound dressing, proliferate and form biofilms. Compared to their planktonic states, the microorganisms contained in the biofilms are more resistant to the ultraviolet (UV) light exposure, disinfectants and a standard dosage of antibiotics, hence require higher concentrations of antibiotics for microbial eradication [6].

An ideal wound dressing material must be biocompatible, biodegradable, and possess anti-infective properties while maintaining a moist environment around the wound site, allowing gaseous exchange, ability to remove excess wound fluid, promote epithelialization and ease of removal without causing additional damage to the wound [7,8].

With the progress in the field of tissue engineering, there is a necessity to develop alternative materials and scaffold techniques to address the requirements. Up to now, numerous strategies have been implemented to fabricate skin scaffolds in the form of electrospun fibers, nano/micro particles (colloidal form), membranes and nanogels [9]. Electrospinning technique remains standalone among all the advanced techniques developed to produce scaffold membranes, due to its simplicity, cost effectiveness, versatility, ease of handling and scalability [10,11]. 

Electrospun nanofibers create an effective physical barrier at the wound region, thereby protect the open wound from further external damages and exogenous microorganisms. Since the structural features of electrospun scaffolds closely mimic the natural extracellular matrix (ECM) [12,13], they can serve as a template for cell adhesion, spreading, and proliferation supporting new tissue regeneration. The electrospun mats allow gaseous exchange, removal/release of wound exudates and maintain a moisturized environment to accelerate the wound healing process [14]. In addition to the above-said advantages, the mats can be loaded with bioactive/therapeutic agents/antimicrobials, which can be released in a controlled manner to the wound site to further promote the wound healing [15]. 

At the present scenario, due to toxicity and multidrug resistance developed by the bacteria against conventional antibiotics, much attention has been given to new classes of antimicrobials [16]. The advanced wound dressings are infused with antimicrobials such as honey [17,18], medicinal plant extracts and their bioactive compounds [19,20,21,22], nanoparticles [23,24,25], essential oils [26,27,28], polycatecholamines [29] and cationic polymers [30]. 

Herbal extracts/phytochemicals have been used from ancient times to cure various diseases and at present in the form of traditional medicine, which is being practiced in many countries [31]. Plant-derived secondary metabolites/phytochemicals serves as the source for developing new potent drugs with fewer side effects [32]. *Gymnema sylvestre* (Asclepiadaceae) commonly employed for the treatment of diabetes, has been reported to have anti-inflammatory and anti-cancer properties and are used to cure obesity, constipation, and improve wound healing [33,34,35,36]. To the best of our knowledge, the *Gymnema sylvestre*’s antimicrobial properties were less exploited [37,38].

Among the wide range of natural and synthetic biodegradable polymers, Poly-ε-caprolactone (PCL) and gelatin were most explored as wound dressing materials. PCL exhibits many advantages such as biocompatibility, biodegradability, excellent processability, and desired mechanical properties [39]. Gelatin has good porosity, biocompatibility, fluid retention properties, cell-specific binding sites and non-antigenicity [13]. However, both the polymers individually are inadequate to fulfil their role as dressing materials due to their certain disadvantages. PCL is hydrophobic, lacks cell-specific recognition sites [40] and degrades at a slower rate [41] whereas gelatin exhibits poor mechanical strength and degradability [31]. In addition, the exposure of electrospun fibers from synthetic polymers to human dermal fibroblasts results in immunogenic response but can be attenuated by co-axial electrospinning with gelatin [42]. Thus, blending PCL with gelatin can produce scaffolds that are mechanically strong and would have cell-specific motifs which would be suitable for accelerated wound healing.

**Scheme 1:**
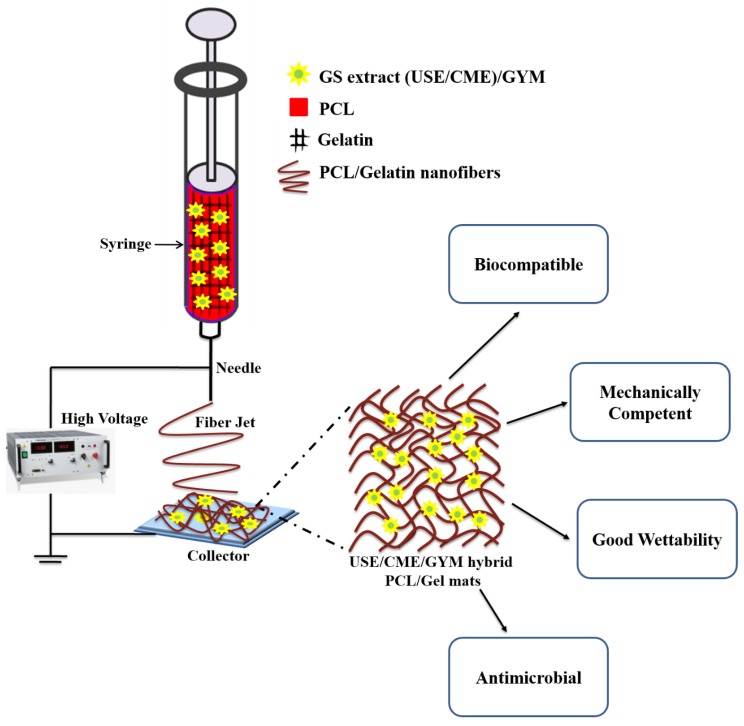
Electrospinning setup used to prepare hybrid mats.

In our previous study, we have developed *Gymnema sylvestre* extract containing PCL nanofibers and investigated their antibacterial and biocompatibility properties [43]. Here, we report the effect of gelatin integration to the PCL/*Gymnema sylvestre* nanofibrous mats, investigated their physical properties, antimicrobial effectiveness and biocompatibility for human primary dermal fibroblasts (hDFs) and cultured keratinocytes (HaCaT cell line). The overall strategy employed to prepare *Gymnema sylvestre* and gymnemagenin infused PCL/Gel wound dressing was shown in Scheme 1.

## 2. Materials and Methods

### 2.1. Materials

Gelatin (type A), Poly-ε-Caprolactone (M_w_ 80,000), Hoechst, 2,2,2-trifluoroethanol (TFE), glutaraldehyde, hexamethyldisilazane (HMDS) and paraformaldehyde were purchased from Sigma Aldrich (Singapore). Dulbecco’s Modified Eagle’s Medium (DMEM) was purchased from Gibco^®^, Thermo Fisher Scientific (Singapore). Other cell culture reagents were obtained from Life Technologies Corporation (Singapore).

#### 2.1.1. Microbial Strains Used

Gram-positive strains: *Staphylococcus aureus* (SA 29213), Methicillin-Resistant *Staphylococcus aureus* (MRSA 700699) and *Staphylococcus epidermidis* (SE 12228).

Gram-negative strains: *Pseudomonas aeruginosa* (PA 9027), and *Escherichia coli* (*E. coli* 8739). All bacterial cultures were from American Type Culture Collection (ATCC, Manassas, VA, USA).

#### 2.1.2. Cell Lines Used

Primary human Dermal Fibroblasts (hDFs) and human keratinocytes cell line (HaCaT) were from American Type Culture Collection (ATCC, Manassas, VA, USA).

#### 2.1.3. Bioactive Compound and Leaf Extracts

Bioactive compound- Gymnemagenin (GYM, purity >95% by HPLC) was bought from Natural Remedies (Bangalore, India).

*Gymnema sylvestre* leaf extracts used in the current study were extracted using two different extraction techniques: ultrasound-assisted extraction (USE) and cold macerated extraction (CME).

### 2.2. Processing of Gymnema sylvestre Leaves

Fresh leaves of *Gymnema sylvestre* were obtained from Tamil University (Tamilnadu, India) and authenticated by scientist Dr. G.V.S Murthy, Southern Regional Centre, Coimbatore, Botanical Survey of India (BSI/SRC/5/23/2016/Tech/215). The methodology for processing the leaves via cold maceration and ultrasound assisted extraction was reported in our previous manuscript [43]. Briefly, the leaf powder was defatted using petroleum ether for 8 h in soxhlet apparatus prior extraction. To obtain cold macerated extracts, 20 g of defatted *Gymnema sylvestre* powder was soaked in 70% methanol (500 mL) for 24 h at 25 ± 2 °C in a rotary shaker. This procedure was repeated thrice and the solvent was filtered, pooled together, concentrated in rotary vacuum at 40 °C and lyophilized into fine powders. To achieve ultrasound assisted extracts, 20 g of powder was soaked in 70% methanol for 3 h and exposed to 40 kHz frequency of ultrasound waves in a digital ultrasonic bath at 50 °C for 50 min. The extracted solvent was filtered, concentrated and made into fine powders as mentioned above. 

### 2.3. Electrospinning of PCL/Gelatin Nanofibers

For electrospinning, PCL (8 wt %) and gelatin (4 wt %) were dissolved separately in TFE, stirred for 5 to 6 h to get a homogenous solution and then mixed together. One hundred microliter of acetic acid was added to the PCL/Gel solution to improve the miscibility. The concentration of CME/USE was 25% and GYM was 0.5% (with respect to *w*/*w* of PCL). CME/USE/GYM was mixed separately to the PCL/Gel solution and stirred overnight. A syringe pump (KDS 100, KD Scientific., Holliston, MA, USA) was used to pump the overnight stirred solution into a 5 mL polypropylene syringe attached to a 23 G needle at a flow rate of 1 mL·h^−1^. To generate electrospun mats, high voltage (Gamma High Voltage Research Inc., Ormond Beach, FL, USA) of 13 kV was applied to the needle tip, which results in the stretching of droplet created at the orifice of the needle and the drawn nanofibers were deposited on aluminum foil wrapped collector which was positioned 13 cm apart from the needle tip [21]. Relative humidity of 60% and a temperature of 22 ± 2 °C was maintained throughout the electrospinning experiments.

### 2.4. Field Emission Scanning Electron Microscopy (FESEM) Analysis

Prior SEM analysis, the prepared nanofibers were sputter coated with platinum to make them conductive using JFC-1600 auto fine coater (JEOL, Peabody, MA, USA). FESEM imaging of as-spun nanofibers was analyzed using JSM-6701F FESEM (JEOL, Peabody, MA, USA) at an accelerating voltage of 10 kV. Image J software (National Institute of Health, Bethesda, MD, USA) was used to calculate the average fiber diameter. Around 50 random fibers were selected for each sample to determine the average diameter.

### 2.5. Fourier Transform Infra-Red Spectroscopy

An FTIR spectrum of different electrospun samples was obtained using Alpha FTIR spectrometer (Bruker GmbH, Ettlingen, Germany). The spectrum was scanned at a resolution of 4 cm^−1^ over the range of 500–4000 cm^−1^ in attenuated total reflectance mode.

### 2.6. Mechanical Properties of Hybrid Mats 

Mechanical properties such as ultimate tensile strength, tensile strain, tensile modulus and toughness of different electrospun mats were determined using tensile tester (Instron 5345, Instron Inc., Norwood, MA, USA). Briefly, nanofibers with an average thickness of 100 µm were cut into rectangular strips (40 × 10 mm). These rectangular strips were tested at a cross-head speed of 10 mm·min^−1^. For each group, samples (n = 4) were tested and the average value was recorded.

### 2.7. Wettability Studies

Sessile drop water contact angle method was adopted to determine the wettability of electrospun mats using VCA optima surface analysis system (AST products, Billerica, MA, USA). Distilled water (1 µL) was used as the solvent to generate droplet on the nanofiber surface. The images were photographed and further processed to obtain the contact angle. For each sample, the testing was conducted in triplicates and the mean ± s.d. values were presented.

### 2.8. Release Kinetics and Scaffold Degradation Studies

The release kinetics studies of *Gymnema sylvestre* loaded mats were conducted by immersing the fiber mats (30 × 30 mm, an average weight of 40.8 ± 5.2 mg) in 3 mL of PBS in triplicate, maintaining them at 37 °C in a shaking incubator. The samples were withdrawn at different time points for analysis by UV spectrometry (OD_292 nm_). The entrapment efficiency was determined by dissolving the electrospun mats (average weight ~20 mg) in TFE, followed by centrifuging at 5000 rpm. The supernatant was collected and analyzed using UV spectrometry (OD_292 nm_). The concentration of the extracts in the release medium was determined from the calibration plot. The experiment was conducted in triplicate.

For scaffold degradation study, the electrospun scaffolds were cut into 2 × 2 cm samples, incubated in PBS at 37 °C for a period of 30 days. At different time intervals, the mats were removed from PBS, washed thrice with milliQ water and dried completely before SEM analysis.

### 2.9. Biocompatibility Studies of USE/CME/GYM Nanofibers

Dermal fibroblasts and keratinocytes play a vital role in various phases of wound healing, hence hDFs and HaCaT cells were selected for assessing the biocompatibility and cell proliferation capability of the prepared mats [44]. Cells were grown as described earlier [45]. For cell proliferation experiments, 8000 cells in 500 µL of medium were seeded into each well of 24 well plates containing various nanofibrous mats (average thickness of 4.3 ± 0.5 µm and weight of 1.6 ± 0.4 mg).

Colorimetric CellTiter 96^®^ AQueous One Solution Cell Proliferation Assay (Promega) was used to determine the cell proliferation of hDFs and HaCaT on the nanofibrous mats. On day 4 and day 7, cells grown on nanofibers were washed twice with phosphate buffer saline (PBS, pH 7.2) to remove debris, non-adherent cells and incubated with DMEM: MTS (4:1) for 3 h at 37 °C. Then the medium was transferred into 96 well plates and the absorbance OD_490 nm_ was measured using microplate reader (BioTek, Singapore). The OD readings were converted into cell number using a calibration curve [46] and average values from two independent triplicate experiments were presented as mean ± s.d.

The hDFs and HaCaT cell phenotype on various nanofibrous mats at day 4 and day 7 were visualized using a laser scanning confocal microscope (Zeiss LSM800, Carl Zeiss Microimaging Inc., Thornwood, NY, USA). Briefly, cells grown on different nanofibrous mats were rinsed with PBS, followed by incubating with paraformaldehyde (4%) for 30 min at room temperature to fix the cells. The fixed cells were then stained with phalloidin (Thermo Fisher Scientific, Singapore) and Hoechst for 1 h to visualize the cell morphologies and nuclei respectively. The stained samples were washed thrice with PBS to remove the excess dyes. Then Flouromount^TM^ (Sigma, Singapore) was used to mount the samples on glass slides and visualized under a confocal microscope using a 40× oil immersion objective lens. Five different spots were imaged for each sample.

Cell morphology of the hDFs and HaCaT on various electrospun mats were analyzed by SEM. Briefly, the cells grown on various mats were washed twice with PBS and 500 µL of 3% glutaraldehyde was added into each well to fix the cells for 30 min. The fixed cells were washed with PBS to remove the glutaraldehyde residues and the samples were dehydrated with a series of ethanol (30% to 100%) followed by 200 µL of HMDS for 5 min. Finally, the dehydrated samples were sputter coated with platinum for visualizing via SEM. 

Collagen secreted by the hDFs after 10 days post seeding on different electrospun mats was determined by Picro Sirius Red staining. Briefly, cells were fixed with 4% formaldehyde, followed by staining with 0.1% Sirius red dye for 1 h. The stained cells were washed with 1× PBS and visualized under an inverted microscope.

### 2.10. Antimicrobial Studies

#### 2.10.1. Radial Disc Diffusion Assay

The antibacterial activity of PCL/Gel+USE/CME mats was assessed using the Kirby-Bauer radial disc diffusion method. Clinical and Laboratory Standards Institute (CLSI) guidelines were followed to carry out the experiments. Initially, the concentration of the bacterial cultures was adjusted to 0.5 McFarland standard, then using a cotton swab the adjusted bacterial cultures were spread uniformly onto the sterile Muller Hinton Agar (MHA, BD, Franklin lakes, NJ, USA) plates. Then PCL/Gel, PCL/Gel+USE, and PCL/Gel+CME mats (25 × 25 mm) weighing about 40.2 ± 4.8 mg were placed onto the center of the MHA plates and incubated at 35 ± 2 °C for 24 h. The zone of inhibition around the mats was photographed and the experiment was conducted in duplicate [47].

#### 2.10.2. Bacterial Cell Viability Assay

Briefly, different electrospun mats (weighing about 40.8 ± 2.5 mg) was placed in 24 well plates and incubated in bacterial suspension (~1 × 10^8^ CFU/mL) for 24 h at 35 °C. One hundred microliter of the culture inoculum was retrieved from each well, serially diluted (10^−1^ to 10^−8^) and plated on a Mueller Hilton agar at 35 °C. The number of viable bacteria persisted in the plates were enumerated using colony counter after 24 h incubation. The experiment was conducted in duplicate.

### 2.11. Statistical Analysis

All the experiments were conducted at least in duplicates and the quantitative data was expressed as mean ± standard deviation (SD). For comparison between groups, one way ANOVA followed by Tukey’s post hoc test was performed. *p* values ≥ 0.05 were considered to be statistically insignificant.

## 3. Results and Discussion

### 3.1. FE-SEM Analysis to Visualise the Surface Morphology and Determining the Fiber Diameter Distribution

**Figure 1:**
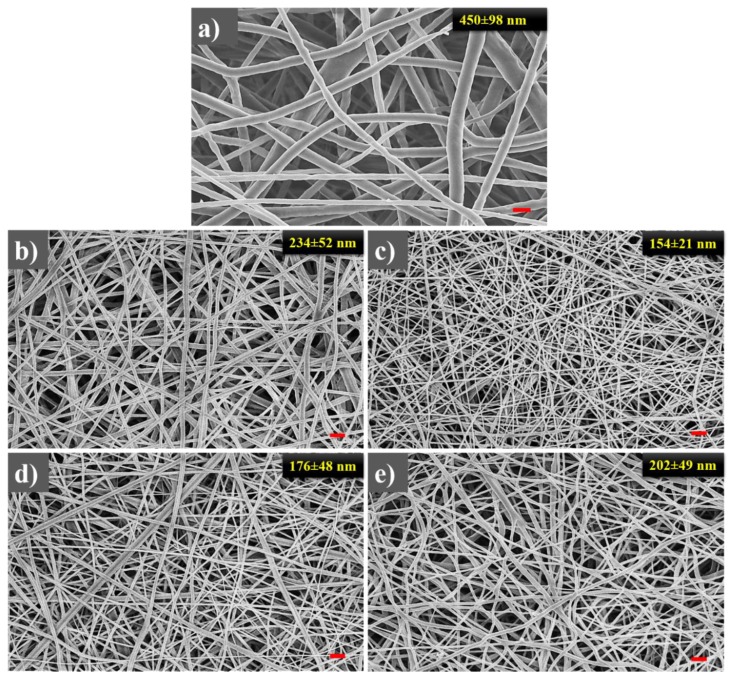
SEM images of electrospun (**a**) Poly-ε-Caprolactone (PCL); (**b**) PCL/Gelatin (Gel); (**c**) PCL/Gel+Ultrasound Assisted Extracts (USE); (**d**) PCL/Gel+Cold Macerated Extracts (CME); and (**e**) PCL/Gel+Gymnemagenin (GYM). Scale bar = 1 µm.

SEM micrographs of the electrospun samples showed bead-free, smooth continuous nanofibers with narrow diameter distribution (Figure 1). The absence of any additional particulate structures and aggregates on the mats clearly ascertain that during the electrospinning process, no phase separation of USE/CME/GYM occurred. The average fiber diameters of PCL, PCL/Gel, PCL/Gel+USE, PCL/Gel+CME and PCL/Gel+GYM were found to be in the range of 450 ± 98 nm, 234 ± 52 nm, 154 ± 21 nm, 176 ± 48 nm and 202 ± 49 nm respectively. The viscosity of the dope solutions containing different extracts was also determined before electrospinning. The solution viscosity of PCL/Gel, PCL/Gel+USE, PCL/Gel+CME and PCL/Gel+GYM were found to be 557 ± 6 cP, 237 ± 8 cP, 260 ± 3 cP and 532 ± 3 cP respectively. The results indicated a substantial decrease in the viscosity of dope solution upon addition of *Gymnema sylvestre* extracts. The obtained values indicate that the addition of *Gymnema sylvestre* (CME/USE) significantly reduced the viscosity of the spinning solution and hence leads to substantial reduction in the diameter of the resultant nanofibers. However, the addition of GYM to the spinning solution did not affect the viscosity and hence there were no significant changes to the diameter of the nanofibers. A similar decrease in the diameter of electrospun synthetic and natural polymers was reported upon addition of natural products such as honey, *G. sylvestre*, henna extracts and attributed to decrease in viscosity of the dope solution [18,43,48].

### 3.2. FTIR Analysis of Composite Mats

**Figure 2:**
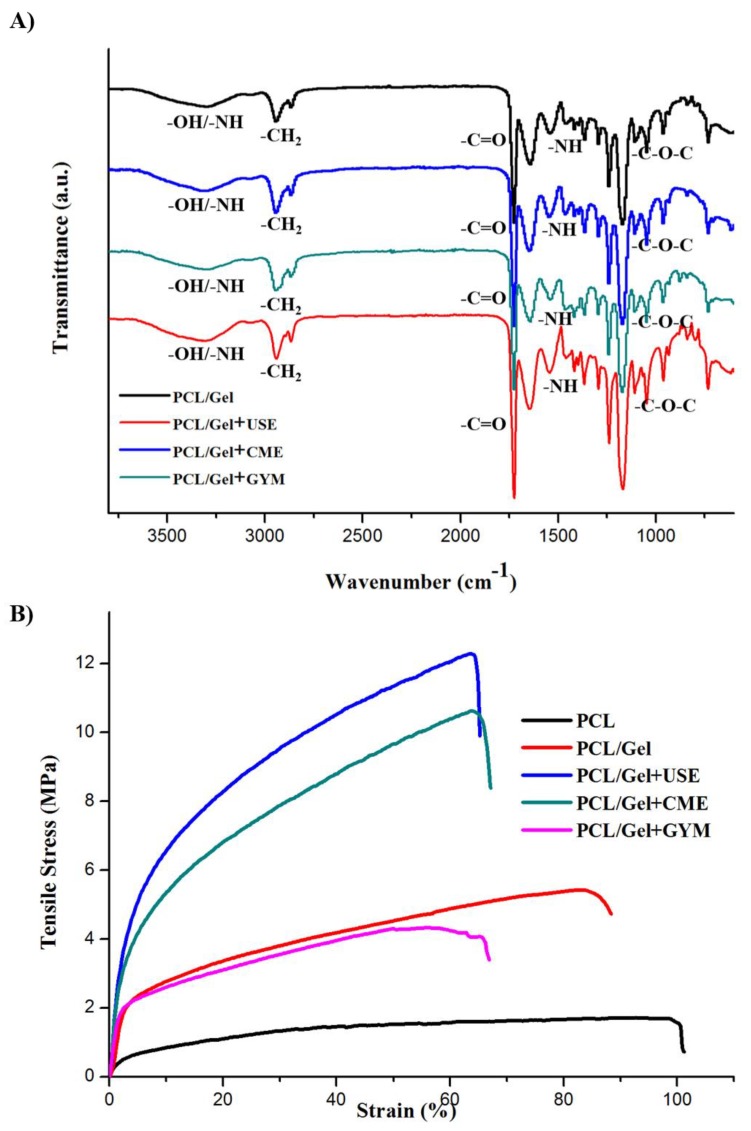
(**A**) FTIR spectrum of PCL/Gel, PCL/Gel+USE, PCL/Gel+CME and PCL/Gel+GYM nanofiber mats. (**B**) Stress-strain curve of PCL, PCL/Gel, PCL/Gel+USE, PCL/Gel+CME and PCL/Gel+GYM nanofibrous mats. For mechanical studies, 4 samples were tested for each mat type (n = 4) and the representative data was shown.

To confirm the integration of CME, USE and GYM into the PCL/Gel mats, the ATR-FTIR spectrum of different electrospun samples was recorded and shown in Figure 2A. The ATR-FTIR spectrum of individual compounds was shown in Figure S1. Characteristic peaks of PCL mat at 2865 and 2946 cm^−1^ correspond to –CH_2_ symmetric and asymmetric vibrations; 1724 cm^−1^ represents C=O stretching of ester group; 1046 and 1240 cm^−1^ related to –C–O–C symmetric and asymmetric stretching vibrations. For gelatin, a peak at 3297 cm^−1^ corresponds to N-H stretching vibration; 1637 cm^-1^ represents the C=O stretching of amide I; 1535 and 1450 cm^-1^ related to the bending vibration of amide II (N–H and –CH_2_); 1240 and 1080 cm^−1^ correspond to the N–H bending and C=O stretching vibration of amide III. The USE/CME extracts revealed –OH stretching at 3316 cm^−1^ related to alcoholic/phenolic groups; C=O stretching of ketones at 1711 cm^−1^ and –C-O stretching of primary and tertiary alcohols at 1034 and 1160 cm^−1^. In gymnemagenin, peaks at 3297, 1603 and 1442 cm^−1^ correspond to –NH (amide) group; C=O stretching vibration of the ester group and –C–C– stretching of aromatic compounds respectively. The characteristic peaks of PCL, gelatin, *Gymnema sylvestre* and gymnemagenin were observed in the electrospun mats, confirming the successful incorporation of the various components into the mats.

### 3.3. Mechanical Properties of Electrospun Hybrid Mats 

The electrospun mats must impart and retain sufficient mechanical support without causing new tissue deformation during wound healing [49]. The stress-strain curves of electrospun PCL, USE/CME/GYM loaded PCL/Gel mats are shown in Figure 2B and Table 1 summarizes the mechanical parameters of different electrospun nanofibers obtained from four independent experiments. 

To infer the effect of *G. sylvestre* extract and gelatin inclusion into the nanofibrous mats, the mechanical properties obtained for different samples were compared against PCL mats. The PCL mats displayed greater plasticity and flexible behavior compared to the other mats. The inclusion of gelatin to the system did not alter the mechanical properties by much. Although, the introduction of USE and CME to the PCL/Gel mats resulted in a decrease in the tensile strain and toughness of the mats, however, a significant increase in the tensile strength and tensile modulus was observed. PCL/Gel+USE laden mats displayed maximum tensile strength and tensile modulus compared to all the samples. However, for PCL/Gel+GYM mats, we did not notice any statistical significant (*p* > 0.05) changes in the mechanical properties.

The mechanical properties of the mats depend mainly on individual fiber microstructure, and, macroscopically on the porosity, and density of inter-fiber bonding sites. In general, for smaller sized fibers the tensile modulus and tensile strength will be higher and for larger sized fibers failure strain will be higher [50,51]. In the case of PCL/Gel+USE laden mats, the fiber diameter (154 ± 21 nm) was smaller and more densely packed compared to that of PCL/Gel fibers (234 ± 52 nm) as shown in Figure 1. Thinner nanofibers create more junction points between them, holding together the adjacent fibers and hence exhibit better mechanical properties compared to the thicker fibers. Moreover, the polar groups of USE/CME were capable of forming intra/inter hydrogen bonding between the fibers, thereby increasing their mechanical properties. Hence the PCL/Gel+USE laden nanofibers possessed superior mechanical strength and sufficient elasticity to function as wound dressing material.

### 3.4. Wettability of Electrospun Mats

**Figure 3:**
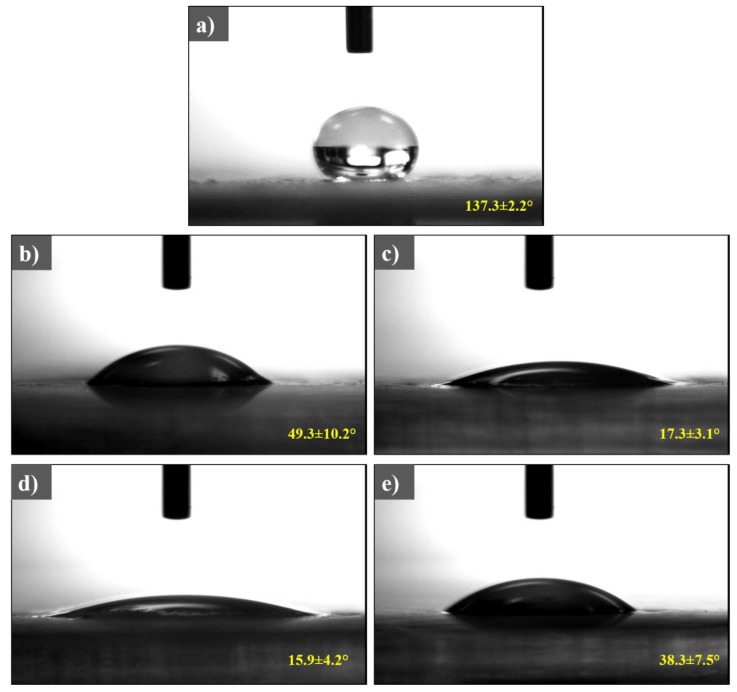
Photographs showing the water contact angle on (**a**) PCL; (**b**) PCL/Gel; (**c**) PCL/Gel+USE; (**d**) PCL/Gel+CME; and (**e**) PCL/Gel+GYM.

Water contact angle measurements have been widely used to determine the hydrophobic effect of water/aqueous solution on surfaces and a useful method to study the effect of additives on surface properties. The biocompatibility and biodegradation of a biomaterial are governed by its surface wettability. To study the effect of gelatin and *Gymnema sylvestre* extracts on the wettability of pristine PCL nanofibers, we determined the contact angles of the mats. Figure 3 shows the images captured on various PCL/Gel mats at 10 s using water droplet to determine the contact angle. The contact angle for PCL, PCL/Gel, PCL/Gel+USE, PCL/Gel+CME and PCL/Gel+GYM were 137.3 ± 2.2°, 49.3 ± 10.2°, 17.3 ± 3°, 15.9 ± 4.2° and 38.3 ± 7.5°, respectively. The increase in the wettability of PCL/Gel mats on adding USE, CME and GYM is due to the presence of polar phytochemicals in the *Gymnema sylvestre* extracts (USE/CME) and availability of the multiple hydrophilic –OH groups in the GYM structure. Wettability of the nanofibrous mats influences the absorption of excess wound exudates and transfer of nutrients. In general, mammalian cells had a better affinity towards the hydrophilic surface compared to its counterpart [52]. Thus the addition of *Gymnema sylvestre* and gelatin to PCL mats enhanced the wettability and making them more suitable for cells to adhere, spread and proliferate.

### 3.5. Release Kinetics and Scaffold Degradation Studies

**Figure 4:**
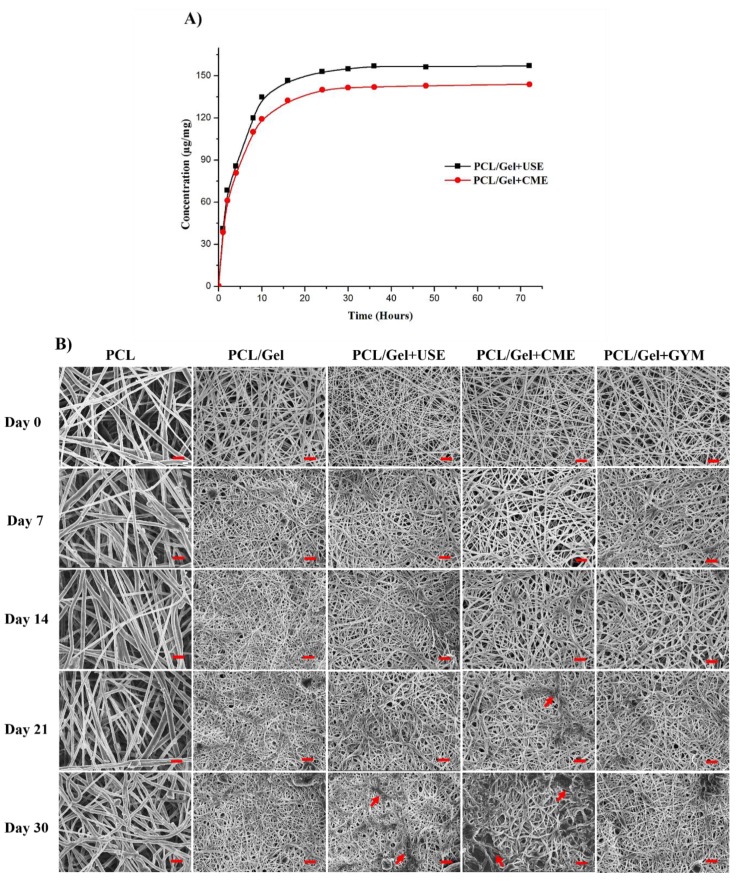
(**A**) Drug release profile of USE/CME from PCL/Gel nanofibrous mats. (**B**) In vitro degradation of electrospun nanofibers.

The release profile of USE/CME mats was investigated to determine whether the active ingredient is released when immersed in buffer. PCL/Gel+USE and PCL/Gel+CME mats contained 187.5 ± 18.6 and 172.0 ± 11.2 µg/mg of *Gymnema sylvestre* leaf extracts, thus having an encapsulated efficiency of 82.5% and 75.8%, respectively. The results indicated a burst release (>50%) of the extracts within the first 8 h after soaking in PBS (Figure 4A). The results are in stark contrast to pristine PCL mats wherein no discernible release of the extract was observed as reported in our previous manuscript [43]. It is likely that extract present over the surface of the nanofibers was released through diffusion, resulting in initial burst release followed by the sustained release from the *Gymnema sylvestre* extracts present at the core of the fiber matrix.

To discern the morphological changes of the mats after immersion in PBS, we imaged PCL, PCL/Gel, PCL/Gel+USE and PCL/Gel+CME mats after soaking for different time duration. Consistent with previous studies, PCL mats immersed in PBS did not show appreciable changes in morphology and remained intact throughout the study (Figure 4B) [53]. However, PCL/Gel or mats containing *Gymnema sylvestre* extracts showed numerous swollen nanofibers with an increasing number of islands of fused fibrous bundles upon immersion in PBS, suggesting possible degradation of the hybrid mats [54]. There was a decrease in the overall porosity of the mats after PBS immersion. It should be noted that due to the presence of PCL, all the mats retained the fibrous structures with fused junctions, further reinforcing the importance of designing hybrid structures that help in enhancing the degradation stability of the wound dressings. Together with the release kinetics studies, the above results suggested that increased wettability and swelling characteristics of the USE and CME mats account for the burst release followed by the controlled release of the extracts.

### 3.6. Cell Proliferation Assessment on the Electrospun Nanofibers

Fibroblasts and keratinocytes are the two major cell types of the skin and play a crucial role in the wound healing process by coordinating with each other to orchestrate a cascade of actions to restore normal tissue functions after injury [55]. To achieve a successful tissue repair, defects at the wound site must be replaced by new granulation tissue followed by the wound closure to restore the physical barrier functions [56]. At the onset of injury, neutrophils are recruited at the wound site to provide the first line of defense against the pathogens followed by monocytes, and macrophages. The growth factors and cytokines released by the neutrophils attracts dermal fibroblasts, which maintain the extracellular matrix [55]. The fibroblasts secrete paracrine factors, such as basic fibroblast growth factor (bFGF/FGF-2) and keratinocyte growth factor (KGF/FGF-7) that promotes keratinocyte growth and differentiation. The keratinocytes, in turn, stimulate the fibroblast to synthesize and crosslink the collagen to fill the damaged ECM. The paracrine signaling also recruits endothelial cells (ECs) which, aid in new vasculature. Finally, the keratinocytes, stratify to form the epithelial layer filling the defect area, thereby facilitating complete wound closure and providing mechanical integrity. The growth factors mediated cross-talk between fibroblasts and keratinocytes during the healing process restores the normal tissue function [57]. To evaluate the biocompatibility, cell proliferation properties of the nanofiber mats, metabolic activity and morphology of the cells (HaCaT and hDFs) were examined at various time points.

#### 3.6.1. MTS Assay

**Figure 5:**
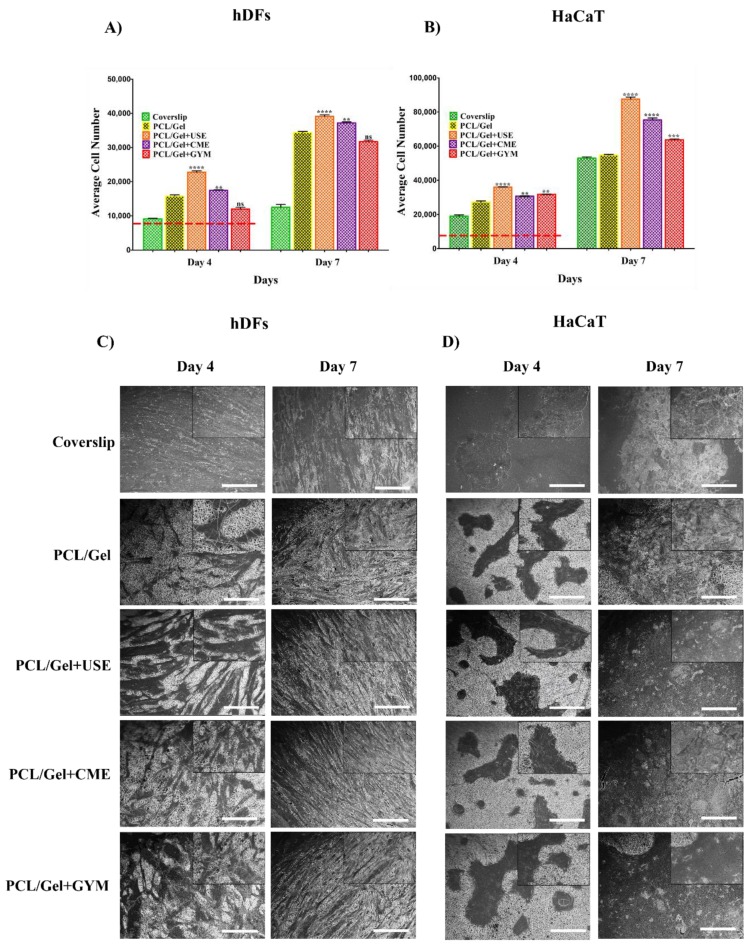
Cell proliferation on electrospun PCL/Gel based mats via MTS assay. (**A**) DFs and (**B**) HaCaT. Statistical significance of cell viability was determined by 1-way ANOVA ** *p* < 0.01; *** *p* < 0.001; **** *p* < 0.0001 and ns *p* > 0.05. SEM images of cells on different electrospun mats. (**C**) DFs and (**D**) HaCaT. Scale bar = 100 µm. Red dotted lines indicate the initial seeding density of cells (8000 cells per well). Insets represent the magnified image (500×).

To confirm the biocompatibility, cell adhesion and cell proliferation of hDFs and HaCaT cells on the PCL/Gel+USE, PCL/Gel+CME and PCL/Gel+GYM nanofibrous mats, they were examined by MTS-based cell viability assay and SEM at day 4 and day 7 post seeding (p.s.). The metabolic activity of seeded hDFs and HaCaT, determined by MTS assay is shown in Figure 5A,B. For hDFs, the cell number increased marginally when seeded on coverslips (used as a control); whereas, increased significantly when seeded on nanofiber mats (Figure 5A). This could possibly due to the availability of more growth space in ECM mimicking 3D nanofibrous matrix compared to the 2D flat coverslip. At day 4 p.s., 2–3 fold increase in hDFs cell density was observed when seeded on PCL/Gel+CME/USE mats. A similar increase in cell density was observed for nanofiber mats at day 7 p.s., as well. Of the four mats, PCL/Gel+USE mats displayed the highest hDFs proliferation when compared to other nanofiber mats. A similar trend was observed for HaCaT cells seeded on nanofiber mats. PCL/Gelatin mats containing the USE displayed the highest HaCaT proliferation when compared to other groups. At day 7 p.s., an about 10-fold increase in cell density was observed on PCL/Gel+USE mats whereas a 6-fold increase was observed for the cells cultivated on coverslips. Thus, the proliferation results confirm that mats loaded with *Gymnema sylvestre* extracts were non-cytotoxic and supported hDFs and HaCaT proliferation, suggesting their excellent biocompatibility for skin tissue engineering. Taking all the results together, the MTS data demonstrated the cell proliferative properties of the *Gymnema sylvestre* loaded mats.

We further analyzed the cell attachment and spreading on different electrospun nanofibers at day 4 and day 7 p.s. by SEM and the images were shown in Figure 5C,D. Even at Day 4 SEM micrograph showed that higher density of hDFs and HaCaT cells attached and spread on the electrospun mats. On day 7, a significant increase in cell number and the confluent layer was observed when seeded on mats containing *Gymnema sylvestre* extracts. The cells looked more spread and densely populated covering the whole area of the mats. The fibroblasts grown on the mats maintained its characteristic elongated spindle-shaped structures whereas HaCaT cells formed a thick layer of microcolonies covering the mats.

#### 3.6.2. F-Actin Staining

**Figure 6:**
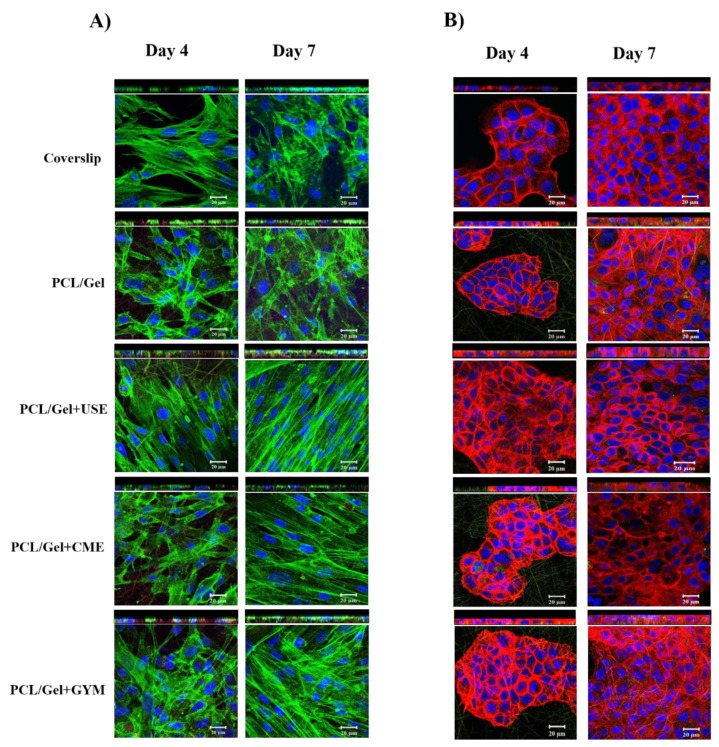
Laser confocal microscopy images on various electrospun mats. (**A**) DFs and (**B**) HaCaT cells. F-Actin stained green for DFs, red for HaCaT and the nuclei were stained blue. Scale bar = 20 µm.

The hDFs and HaCaT cell phenotypes were observed by staining of cell cytoskeleton and nucleus, imaged by confocal microscopy (Figure 6). A spindle-shaped morphology was clearly visualized for hDFs with intact cytoplasmic filamentous distribution (green) of F-actin and prominent nucleus (blue) with no structural abnormalities. Notably, for fibroblasts seeded onto PCL/Gel+USE and PCL/Gel+CME, increased alignments of the cells were observed as indicated by the higher aspect ratio of the nuclei at day 7 post seeding. Bashur et al. reported that an increased diameter and degree of orientation of electrospun fibers contributed to the enhanced adhesion and the aspect ratio of fibroblasts [58]. Together with the increased wettability and swelling of PCL/Gel+USE and PCL/Gel+CME mats after immersion in PBS, it is likely that the increased diameter of the nanofibers may contribute to the observed alignment of hDFs. For HaCaT cells, characteristic microcolonies were observed with F-actin stained red and nucleus in blue. The results of F-actin staining corroborates well with SEM micrographs, as day 4 images clearly revealed scattered well-aligned cells attached to the mats. The day 7 micrographs showed a confluent layer of cells spread throughout the mats. Z-section analysis revealed the thickest cell layers for PCL/Gel+USE for both cell lines at day 7. Overall, these results illustrated that PCL/Gel+USE mats could increase the attachment and proliferation of HaCaT and hDFs, further confirming the excellent biocompatibility of the mats.

#### 3.6.3. Collagen Expression

Finally, the expression of ECM by the cells seeded on various scaffolds was determined by picro-sirius red staining for collagen expression. Figure S2 showed images of the scaffolds that were stained by picro sirius red stain for the expression of collagen by hDFs at Day 10 p.s. Among all the mats, PCL/Gel+USE containing mats exhibited a uniform distribution of red colouration whereas sporadic staining was observed for PCL/Gelatin and PCL/Gel+CME mats, suggesting that hDFs cultivated on PCL/Gel+USE mats produced a uniform distribution of the ECM. Collagen type I is one of the most abundant proteins of the human body which constitutes the majority of ECM. The structure of collagen consists of a triple helical structure of fibrils aligned in different orientations. Attachment of cells to collagen allows for various essential mechano-transduction signaling cascades to occur within the cell, as well as the creation of an avenue for cell migration due to the chemotactic role of collagen. During the remodeling phase of wound healing, fibroblasts secrete collagen which plays a critical role as their interwoven fibrils replace the provisional fibrin-based matrix, imparting improved mechanical strength and aiding wound contraction [21].

Taken together, the bioactive compounds present in *Gymnema sylvestre* could be a possible reason for better adherence and proliferation of the hDFs and HaCaT. The major bioactive compounds present in *Gymnema sylvestre* extracts were gymnemic acids and gymnemagenin [59,60,61]. The other phytoconstituents from *Gymnema sylvestre* reported were kaempferol, quercetin [62], triterpenoid saponin [63], lupeol and stigmasterol [64]. All the above-mentioned cell culture experiments confirmed that the *Gymnema sylvestre* and GYM loaded PCL/Gel mats were not toxic and promote the proliferation of hDFs and HaCaT, with PCL/Gel+USE laden mats demonstrating their excellent biocompatibility among all the mats.

### 3.7. Antibacterial Activity of Electrospun Gymnema Sylvestre Mats

The important prerequisite for modern wound dressings is to prevent the bacterial infection at the wound site from resident and external sources. Kirby-Bauer disc diffusion assay was used to assess the antimicrobial activity of USE/CME loaded PCL/Gel mats. As expected no zone of inhibition was observed around the electrospun PCL/Gel mats whereas mats containing the *Gymnema sylvestre* extracts displayed a clear zone of inhibition (Figure S3). The zone of inhibition in mm for different strains was shown in Table 2. The values are lower for Gram-negative bacterial than Gram-positive bacteria, possibly due to the presence of an additional outer membrane in the Gram-negative bacteria. Consistent with these results, MIC determination by broth dilution suggested 2–3 fold higher MIC values against the tested *E. coli/P. aeruginosa* strains.

**Figure 7:**
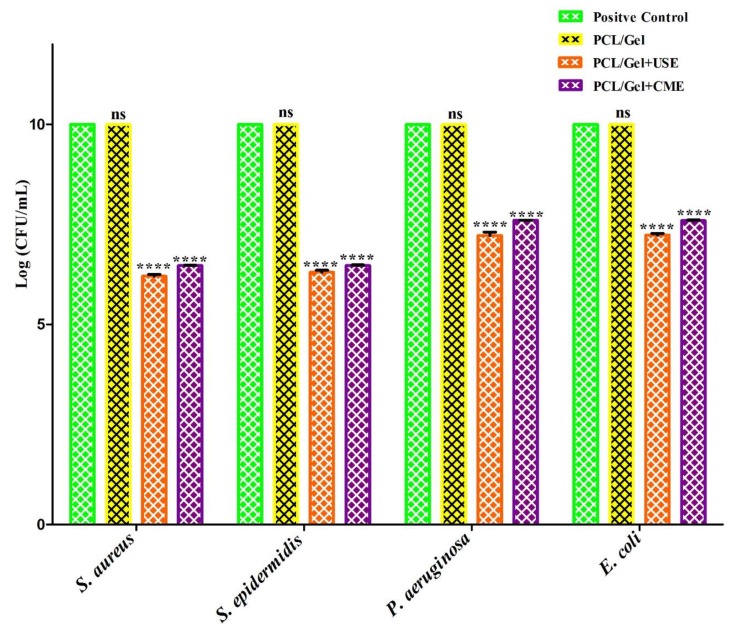
Bactericidal activity of electrospun mats against microorganisms. Statistical significance compared against positive control determined by 1-way ANOVA **** *p* < 0.0001 and ns *p* > 0.05.

The antibacterial activity of the electrospun mats was further evaluated using bacterial cell viability assay under proliferating conditions. Figure 7 shows the bacterial survivors expressed in terms of log CFU/mL values obtained for microbes exposed to various electrospun mats. For Gram-positive bacteria, PCL/Gel+USE and PCL/Gel+CME mats displayed > 2 log reduction (>99% decrease in bacterial viability) and for Gram-negative bacteria, they displayed a >1 log reduction (>90% decrease in bacterial viability) when compared to initial inoculum, whereas PCL/Gel mats did not display any antimicrobial activity. It should be noted that there was a significant decrease in the bacterial inoculum after exposure to PCL/Gel+USE and PCL/Gel+CME when compared to initial bacterial inoculum. Together with the disc diffusion assay, these results confirmed the potent bactericidal properties of PCL/Gel+USE/CME mats.

## 4. Conclusions

The present study demonstrated the utility of electrospun PCL/Gelatin mats containing antimicrobial *Gymnema sylvestre* extracts for skin tissue engineering. The inclusion of gelatin to the PCL/*G. sylvestre* system resulted in increased wettability and allowed the extract to leach out from the fibers. PCL/Gel+USE loaded mats possess all pre-requisite physical and biological properties to encourage the attachment, spreading and proliferation of the fibroblasts and keratinocytes with potent antimicrobial properties against commensal pathogens such as *S. aureus* or *S. epidermidis*. The initial burst release of extracts from the electrospun mats effectively could avert the bacterial colonization of the injured tissue, thereby eliminating the threat of infection which delays the healing process. The degradation study inferred that these hybrid PCL/Gel mats were structurally stable, thereby reducing the frequency of dressings and nursing costs. In conclusion, we have demonstrated a simplistic approach for the preparation of *Gymnema sylvestre* loaded PCL/Gel hybrid mats and their feasibility as a nanofibrous anti-infective wound dressing in near future.

## Figures and Tables

**Table 1:** Mechanical properties of different electrospun mats. Statistical significance of mechanical properties was calculated by 1-way ANOVA * *p* ≤ 0.05; *** *p* < 0.001; **** *p* < 0.0001 and ns *p* > 0.05.

Sample	Tensile Modulus (MPa)	Ultimate Tensile Stress (MPa)	Ultimate Tensile Strain (%)	Toughness (MJ·m^−3^)
PCL	5.58 ± 0.86	1.5 ± 0.21	100.5 ± 12.5	10.81 ± 3.25
PCL/Gel	12.52 ± 2.84 ^ns^	4.9 ± 0.58 ^ns^	75.67 ± 9.06 ^ns^	2.33 ± 0.92 ^ns^
PCL/Gel+USE 25	76.32 ± 12.3 ****	10.44 ± 1.56 ***	69.78 ± 7.6 ^ns^	6.01 ± 0.5 ^ns^
PCL/Gel+CME 25	41.73 ± 9.57 ***	8.93 ± 1.21 ***	58.36 ± 12.16 ^ns^	5.22 ± 1.19 ^ns^
PCL/Gel+GYM	11.6 ± 0.47 ^ns^	5.88 ± 2.4 *	64.34 ± 5.52 ^ns^	3.65 ± 1.21 ^ns^

**Table 2:** Antibacterial activity of USE/CME loaded PCL/Gel nanofibers.

Microorganism	Zone of Inhibition (mm) Excluding Fiber Diameter	
	PCL/Gel+USE	PCL/Gel+CME
*Staphylococcus aureus* 29213	15.2 ± 3.6	7.6 ± 2.2
Methicillin-Resistant *Staphylococcus aureus* 700699	10.4 ± 1	5.2 ± 1.7
*Staphylococcus epidermidis* 12228	18.5 ± 2.2	10.6 ± 1.5
*Pseudomonas aeruginosa* 9027	6.3 ± 1.1	3.1 ± 0.3
*Escherichia coli* 8739	4.2 ± 0.8	1.9 ± 0.2

## Supplementary Materials

The following are available online at https://www.mdpi.com/2079-4991/9/3/462/s1, Figure S1: FTIR spectrum of individual compounds, Figure S2: Collagen staining on various nanofibrous scaffolds, Figure S3: Disc diffusion images of *Gymnema sylvestre* loaded PCL/Gel mats respectively.
