# Supplementary material for: Poly-ε-Caprolactone/Gelatin Hybrid Electrospun Composite Nanofibrous Mats Containing Ultrasound Assisted Herbal Extract: Antimicrobial and Cell Proliferation Study
Source: Nanomaterials (Basel). 2019 Mar 20;9(3):462. doi: 10.3390/nano9030462 (PMC6474082; doi:10.3390/nano9030462)
Supplement: Supplementary file 1 [file nanomaterials-09-00462-s001.pdf]

Supplementary Information

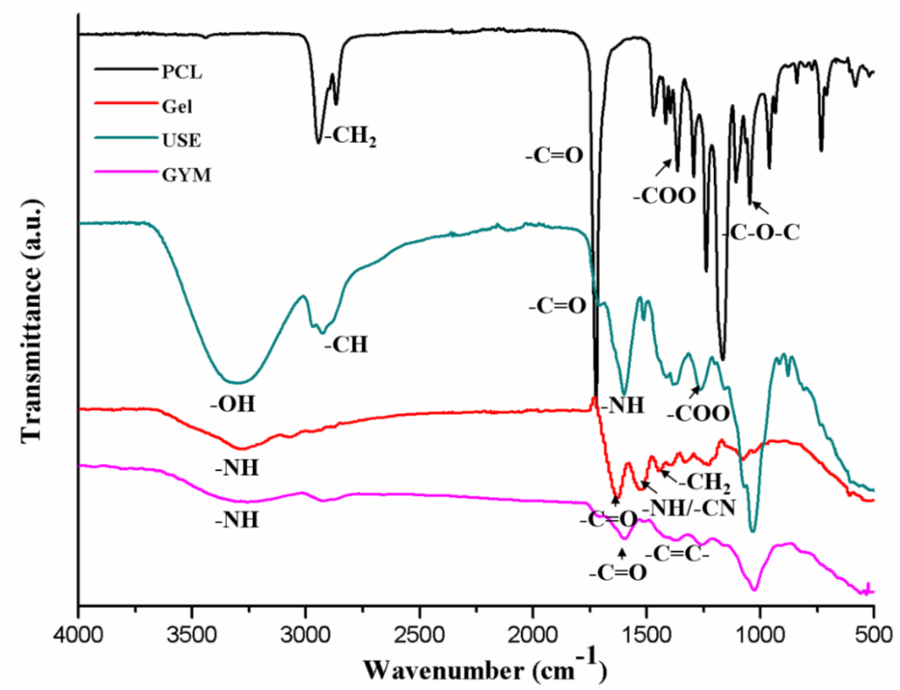

Figure S1: FTIR spectrum of PCL, Gel, USE, and GYM.

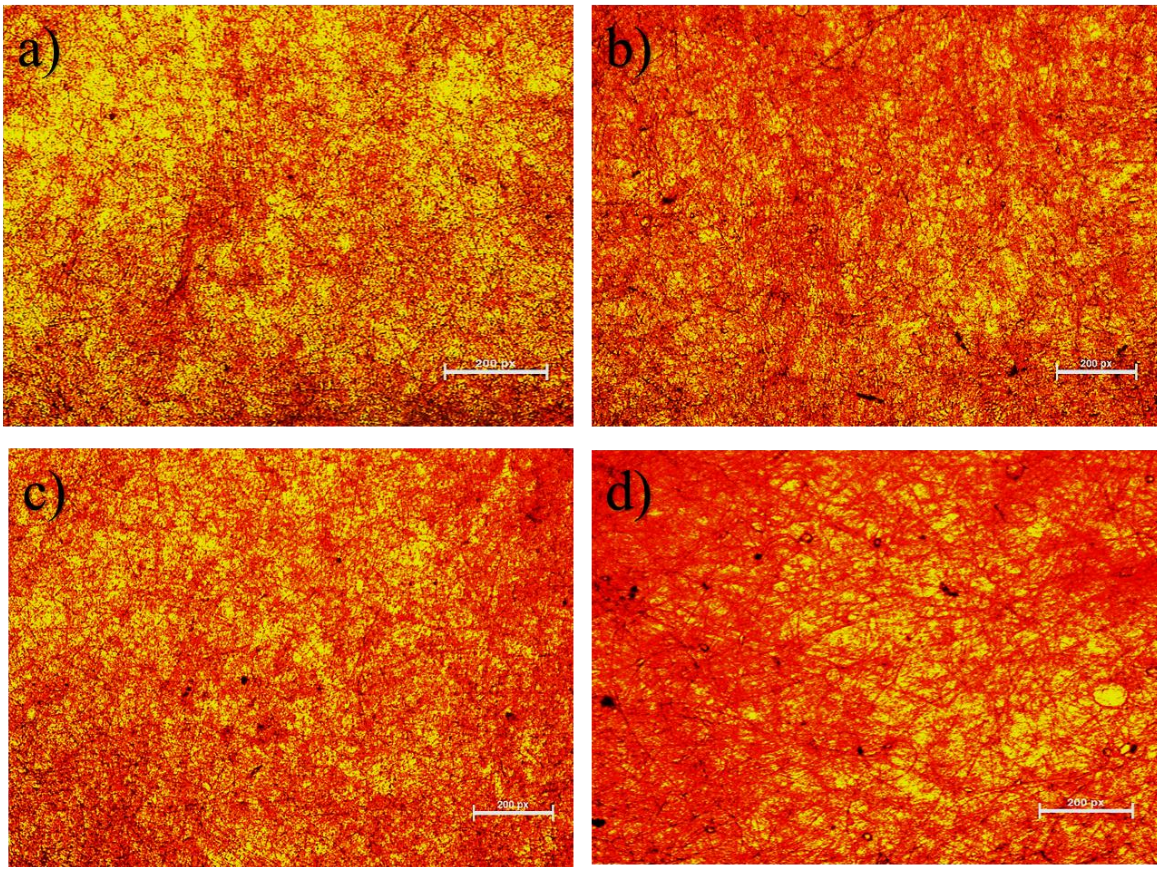

Figure S2. Collagen staining on various nanofibrous scaffolds a) PCL/Gel, b) PCL/Gel+USE, c) PCL/Gel+CME, and d) PCL/Gel+ GYM. Scale bar = 200 μm.

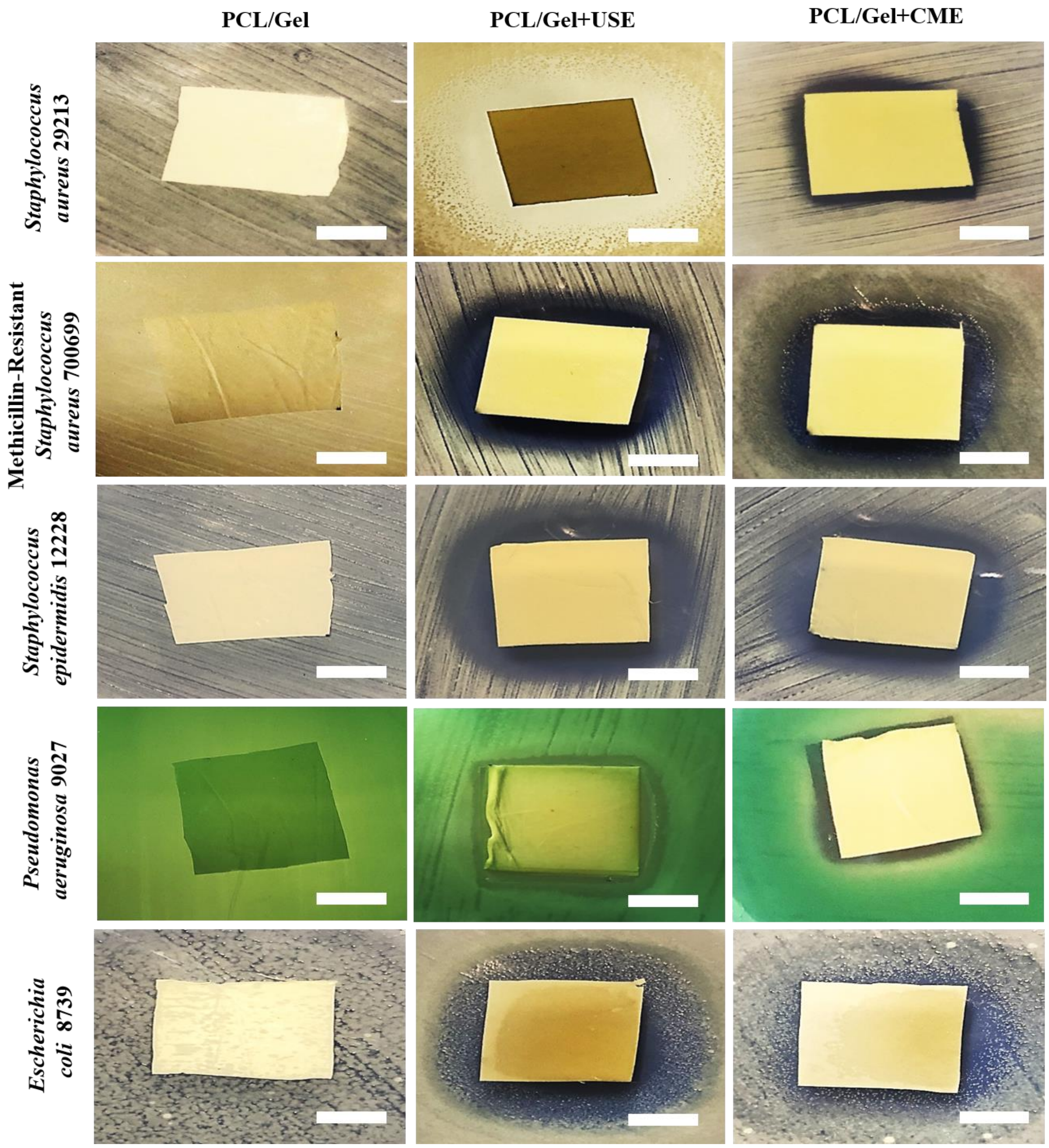

Figure S3: Disk diffusion images of *Gymnema sylvestre* loaded PCL/Gel mats against the microorganisms. Scale bar = 10 mm.
